# Information provision for orthognathic treatment by consultant orthodontists in the United Kingdom and Republic of Ireland: A questionnaire-based study

**DOI:** 10.1177/14653125251391432

**Published:** 2025-11-26

**Authors:** Robert SD Smyth, Fiona S Ryan, Sophy K Barber, Susan J Cunningham

**Affiliations:** 1 Orthodontic Unit, University College London Eastman Dental Institute, London, UK; 2 Orthodontic Department, Royal National ENT and Eastman Dental Hospitals, University College London Hospitals NHS Foundation Trust, London, UK; 3 Orthodontic Department, University of Leeds, Leeds, UK

**Keywords:** orthognathic treatment, decision-making, information provision, risks, benefits

## Abstract

**Objectives:**

The aim of this research was to carry out a study of consultant orthodontists in the United Kingdom (UK) and Republic of Ireland (RoI), exploring the information provision for patients both considering and undergoing orthognathic treatment to help inform the development of decision support tools.

**Design:**

A questionnaire-based study of consultant orthodontists in the UK and RoI through the British Orthodontic Society (BOS) Consultant Orthodontist Group.

**Setting:**

Data collected using an online survey platform (Qualtrics^®^ XM).

**Results:**

A final sample of 56 respondents was achieved with an estimated response rate of 28%. All respondents (100%) said their patients routinely attended an orthognathic multidisciplinary team (MDT) clinic before commencing any active treatment. The majority (98.21%) of respondents said they utilised nationally available resources produced by the BOS, with the most commonly used resource being the BOS ‘*Your Jaw Surgery*’ online resource. When considering the benefits of treatment that were routinely discussed, the three most commonly selected responses were improvement in occlusion (92.86%), improvement in facial aesthetics (91.07%) and improvement in dental aesthetics (83.93%). There were nine orthodontic risks that were routinely discussed by more than 90% of respondents, including pain and discomfort, duration of treatment and long-term commitment to retainer wear, alongside 11 surgical risks, including permanent and temporary paraesthesia or dysaesthesia of the lips/chin/tongue, and postoperative pain.

**Conclusion:**

From the responses received, patients considering orthognathic treatment routinely attend an orthognathic MDT clinic before commencing active treatment. Information is provided with nationally available resources and consultant orthodontists routinely discuss risks and benefits of the orthodontic and surgical treatment with prospective patients to help them make informed decisions regarding their care.

## Introduction

Orthognathic treatment is a complex, elective procedure that may be considered when there is a severe dentofacial discrepancy that cannot be corrected with orthodontics alone (Proffit et al., 2003). To facilitate the surgical procedure, the traditional approach is to carry out orthodontic treatment first to ensure the teeth are positioned optimally to allow surgical correction. After the surgical procedure, a period of postoperative orthodontics is also required to settle the occlusion. However, surgery first can be considered in certain patients. In the UK, the majority of orthognathic treatment is undertaken in a National Health Service (NHS) hospital setting. The Republic of Ireland (RoI) has a similar setting for orthognathic care. Multidisciplinary team (MDT) clinics are commonly utilised, where specialists from all relevant fields collaborate to provide comprehensive care for patients. These clinics typically include orthodontists, oral and maxillofacial surgeons, psychiatrists/psychologists and other healthcare professionals who work together to address the diverse needs of patients.

Shared decision-making and providing adequate information to patients is one of the major keys to patient satisfaction with outcomes of treatment and is considered a foundation for overall treatment success (Akram et al., 2021; Alkharafi et al., 2014; Brouns et al., 2022; Charles et al., 1997; Graf et al., 2022; Pachêco-Pereira et al., 2016; Paul et al., 2022; Ryan and Cunningham, 2014; Watts et al., 2018). As a result of the Montgomery vs Lanarkshire ruling, clinicians are now required to provide patients with the details of all material risks to which ‘a reasonable person in the patient’s position would attach significance to the risk’ (United Kingdom Supreme Court, 2015).

Information can be provided to patients using a variety of methods. Traditionally, a description of the procedure to be undertaken and the risks and benefits would be carried out as a verbal discussion between the patient and clinician(s). Written information leaflets have been developed to supplement this, both locally and on a national basis, and may be available in paper format and/or electronically. Further to traditional methods of information delivery, new styles of clinic, such as patient and family information clinics, have been developed to support the traditional orthognathic consultation. These have been shown to increase involvement in the decision-making process and enhance patient satisfaction (Bergkulla et al., 2017; Ryan et al., 2011). At present, there are only a small number of units in the UK that employ this style of clinic for their patients and details regarding these clinics are available in previous publications (Akram et al., 2021; Ryan et al., 2011).

Patients can also access vast amounts of information via the Internet. In 2015, the British Orthodontic Society (BOS), the national association for orthodontists in the UK, developed an online resource for patients on its website; this is called ‘*Your Jaw Surgery*’ and contains four sections: Patient Journey, Patient Stories, Your Surgery Explained and Other Resources. Patients may also access information via other websites, such as hospital or practice websites, other professional bodies, patient forums and blogs, Twitter, YouTube and other forms of social media. Clinicians should exercise care when making recommendations around specific websites, as many have been found to be of low validity and quality (Aldairy et al., 2012; Engelmann et al., 2020). A recent review of 46 YouTube videos relating to orthognathic surgery found they were of poor quality overall. Using the validated DISCERN tool (Charnock et al., 1999) that assesses the quality of written healthcare information for treatment choices, the mean score for the overall quality of the videos was 14.2, which falls into the ‘very poor’ category of DISCERN scores (score range = 16–28) (Puthumana et al., 2022).

Although studies have shown satisfaction with information provided in various formats (Akram et al., 2021; Al-Hadi et al., 2019; Graf et al., 2022; Ryan et al., 2011), to date no assessment has been undertaken in the UK or RoI regarding what information is provided to patients and at what stage(s) in the patient journey.

### Aims

The aims of the present study were therefore to determine: (1) whether patients are being provided with information about orthognathic treatment before attending their first MDT clinic; (2) what type of information is provided or recommended to orthognathic patients; (3) if patients are directed to resources developed by the BOS, such as information leaflets or the BOS ‘*Your Jaw Surgery*’ online resource; and (4) what benefits and risks are discussed with patients and at what stage in the patient journey.

## Methods

A bespoke, web-based, questionnaire was developed by the research team, all of whom had experience of treating patients undergoing orthognathic treatment and of working in orthognathic MDTs. Questions were developed based on an in-depth review of the literature and the experiences of the team and colleagues. There were four drafts of the questionnaire, with each draft being reviewed and critically appraised by all members of the research team and amendments made before review of the next draft. The questionnaire (Supplementary Figure 1) was hosted on the Qualtrics^®^ XM (Provo, UT, USA) platform and consisted of five sections:

- About you
- When do you provide information to prospective patients?
- What information do you provide?
- Use of national resources
- Risks and benefits discussed

A pilot study was conducted involving four consultant orthodontists based in different geographic areas of the UK. This was a convenience sample of clinicians known to the authors and who have experience of treating orthognathic patients. Readability of the questionnaire was assessed using the Flesch Kincaid Reading Ease score. The score of 39.3 is at a ‘College’ reading level and the authors felt this was appropriate for the target audience in this study of professionals with postgraduate degrees. After amendments and minor formatting changes, ethical approval was obtained on 21 December 2023 from the University College London (UCL) Research Ethics Committee (Project ID: 10569/002). Approval was also obtained from the Clinical Governance Directorate of the BOS for distribution of the questionnaire to members of the BOS Consultant Orthodontist Group (COG) via the BOS administrative staff. The inclusion criterion was consultant orthodontists working in the UK and RoI who personally treated orthognathic patients using the conventional approach. The questionnaire contained a link to the participant information sheet, and the consent form was included at the start of the questionnaire. The first email was sent on 5 March 2024, with a follow-up on 19 March 2024. The study was also discussed at the business meeting of the BOS COG Symposium in March 2024 by a member of the research team. The questionnaire was closed to responses on 9 April 2024 after 5 weeks of data collection.

All descriptive analyses were carried out by two researchers (RSDS and SJC) using Microsoft Excel for Mac version 16.83 (Microsoft, 2024). Data were handled in line with GDPR 2018 and the Data Protection Act 2018. This study was conducted in accordance with the Strengthening the Reporting of Observational studies in Epidemiology (STROBE) guidelines for cross-sectional studies. A completed STROBE checklist is available in the supplementary materials (von Elm et al., 2008) (Supplementary Figure 2).

## Results

In total, 67 responses were received. Of these, the data provided by three respondents were excluded because they did not personally treat routine orthognathic patients. A further eight respondents were excluded because they did not complete the questionnaire in full. A final sample of 56 responses was therefore achieved. The number of consultant members of the COG is estimated at 200, giving an approximate response rate of 28%.

### Respondent characteristics

All 56 respondents said they did personally treat orthognathic patients. Most respondents worked in England (87.50%) (Table 1).

**Table 1.** Respondents’ main country of work.

Country	n (%)
England	49 (87.50)
Scotland	4 (7.13)
Wales	1 (1.79)
Northern Ireland	1 (1.79)
Republic of Ireland	1 (1.79)
Total	56 (100)

The median time practising as a consultant orthodontist was 18.5 years, with the shortest time being 1 year and the longest 40 years (interquartile range = 13–23). All respondents said their patients routinely attended an orthognathic MDT clinic before commencing any active treatment. The majority of respondents (71.42%) attended one or two orthognathic MDT clinics a month.

### Timing of information provision

The majority of respondents (87.50%) reported that they provide an overview of the likely timescale of orthognathic treatment at the initial new patient consultation. When it came to providing detailed information that would allow someone to make a fully informed decision about whether to proceed with treatment, the most commonly selected option was at the first multidisciplinary clinic before any treatment starts (51.09%). It was possible to select multiple responses, as the information may be discussed and revisited over multiple appointments. Detailed information was provided at the initial new patient appointment by 17.39% of respondents and at the time of orthodontic treatment consent by a similar number (15.22%).

### Information provision

**Figure 1.**
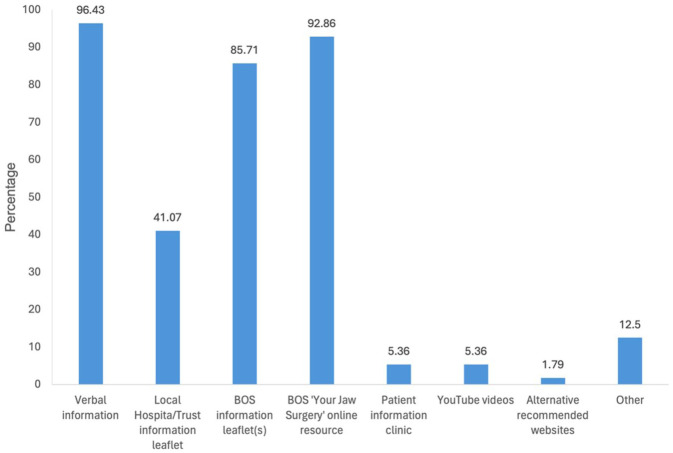
What type of information do you provide or recommend to patients considering orthognathic treatment? NB: Respondents could select more than one option so numbers do not add up to 100%.

With respect to the type of information provided to patients considering orthognathic treatment, the most frequently selected responses were verbal information (96.43%), the BOS ‘*Your Jaw Surgery*’ online resource (92.86%) and BOS information leaflets (85.71%). Figure 1 shows the distribution of responses regarding the types of information provided.

Four respondents (7%) reported that their hospital ran a patient information clinic, where multiple patients and friends/family can attend together to receive information from the team. To avoid double counting of hospitals, respondents were asked to state which hospital they work in. Responses were received for two hospitals that have historically carried out this type of clinic. The responses received from the two hospitals detailed that patients attend a patient information clinic before active treatment commences.

### Use of national resources

Of the respondents, 98.21% said they used nationally available resources produced by the BOS. The most commonly used resource was the BOS ‘*Your Jaw Surgery*’ online resource (n = 53, 94.64%), followed by hard copy information leaflets (32.12%) and digital information leaflets via a QR code (29.20%).

When asked which additional resources or tools would be useful to support patients during the decision-making process (Table 2), the most commonly selected response was discussion with a past patient (73.21% of respondents), followed by support from a mental health professional as part of the multidisciplinary team on the orthognathic clinic (71.42%) or within the hospital by referral (58.92%). A patient decision aid was also selected by 35 (62.5%) respondents. When asked at which time points respondents thought resources would be most helpful, the most selected option was after the multidisciplinary clinic but before active treatment commences (73.21%), followed by before their first multidisciplinary clinic (during the records phase) and during the first multidisciplinary clinic, which were both selected by 39.29% of respondents.

**Table 2.** Additional forms of support that may be useful for patients during their decision-making process.

Information resource	n (%)
Discussion with a past patient(s)	41 (73.21)
Support from a mental health professional (psychiatrist/psychologist) as part of the multidisciplinary team in your orthognathic clinic	40 (71.42)
A patient decision aid	35 (62.50)
Support from a mental health professional (psychiatrist/psychologist) within the Trust by referral	33 (58.92)
Access to a patient information clinic	16 (28.57)
Decision coaching from a trained professional	9 (16.07)
I do not think any of the above are necessary	2 (3.57)
Other	1 (1.79)

Respondents could select more than one option so numbers do not add up to 100%.

**Figure 2.**
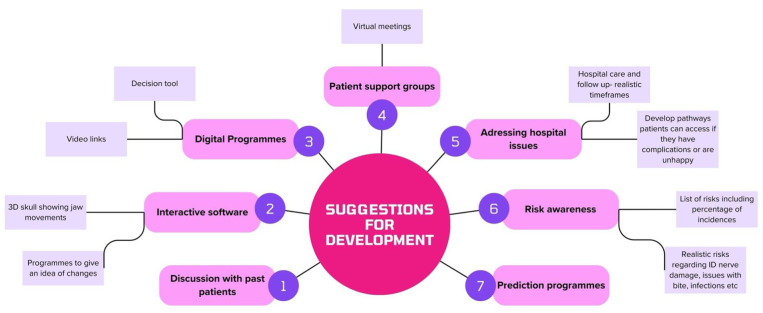
Summary of suggestions received for development of new methods of information provision for orthognathic treatment.

In total, 31 (55.36%) respondents thought it would be useful to develop further methods of information provision for orthognathic treatment patients. A free text box was available, and respondents were encouraged to enter any suggestions they had. There were 21 suggestions made, for which the free text topics were summarised into themes in a basic content analysis, some of which are detailed in Figure 2.

### Discussion of benefits and risks

#### Benefits

**Figure 3.**
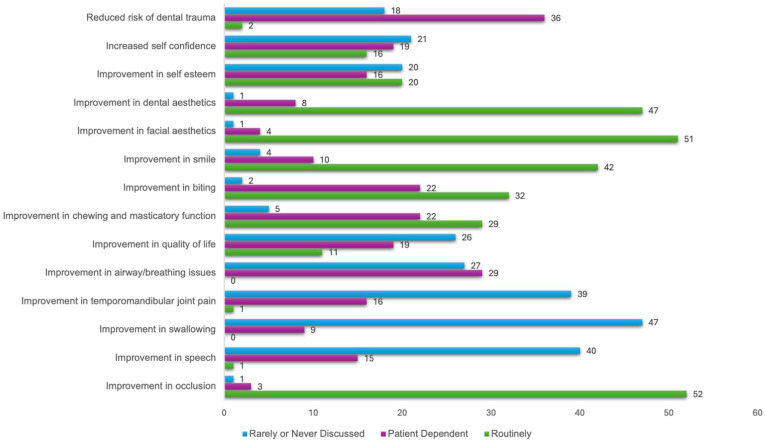
Which benefits do your team discuss with patients considering orthognathic treatment. NB: Number of responses shown.

When considering benefits that were ‘routinely’ discussed (Figure 3), the three most commonly selected responses were improvement in occlusion (92.86%), improvement in facial aesthetics (91.07%) and improvement in dental aesthetics (83.93%). When considering benefits that were ‘rarely or never discussed’, the three most commonly selected responses were improvement in swallowing (83.93% of respondents reported rarely or never discussing this), improvement in temporomandibular joint (TMJ) pain (69.64%) and improvement in airway/breathing issues (48.21%).

#### Orthodontic risks

**Figure 4.**
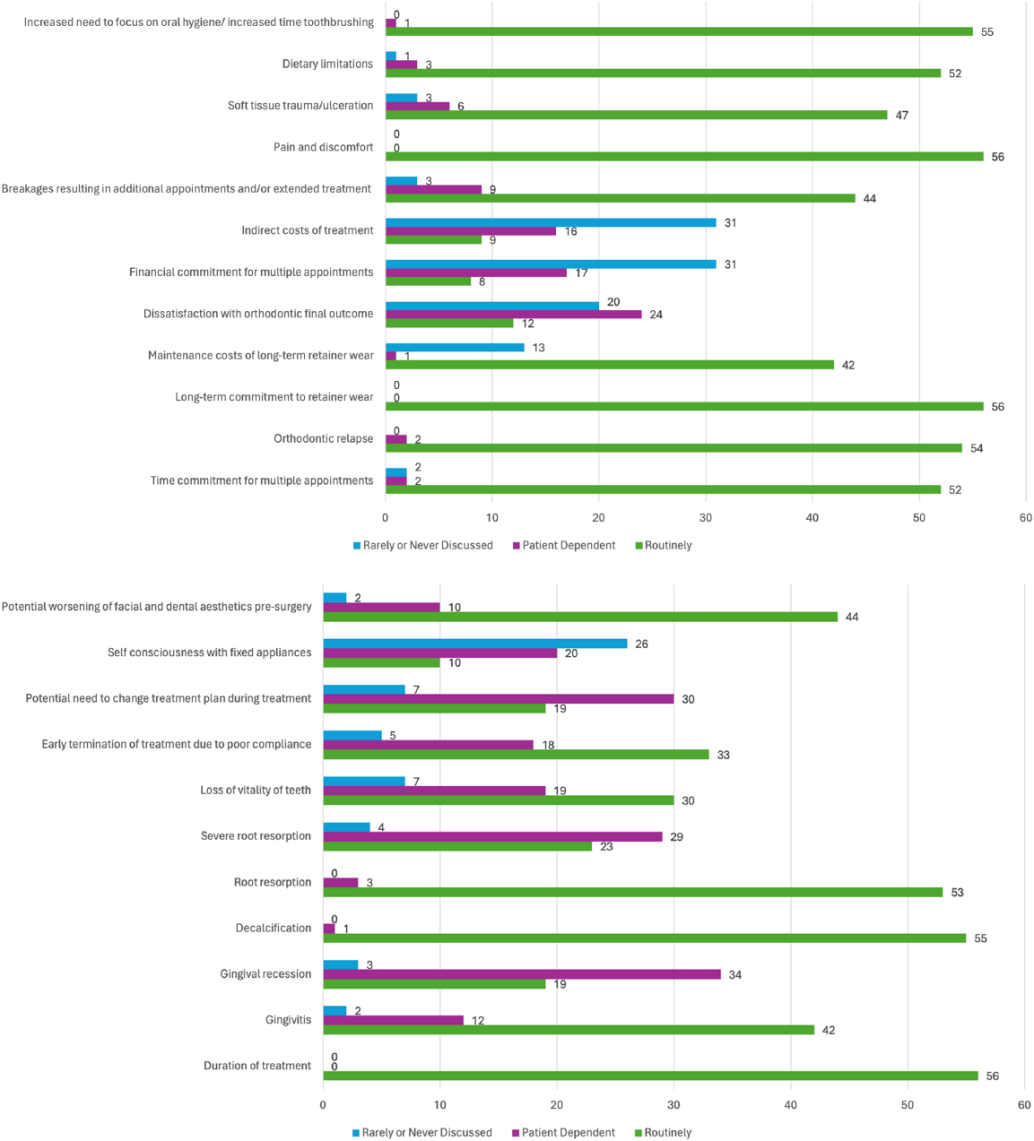
Which orthodontic risks and other potentially negative aspects of treatment do your team routinely discuss with patients considering/undergoing orthognathic treatment? NB: Number of responses shown.

There were three risks, or ‘downsides’, to treatment, that were ‘routinely’ discussed by all 56 respondents (100%) (Figure 4). These were pain and discomfort, duration of treatment and long-term commitment to retainer wear. Alongside this, a further six risks were ‘routinely’ discussed by more than 90% of respondents. These were decalcification (98.21%), increased time to focus on oral hygiene/increased time toothbrushing (98.21%), orthodontic relapse (96.43%), root resorption (94.64%), time commitment for multiple appointments (92.86%) and dietary limitations (92.86%).

The options which were selected as being ‘rarely or never discussed’ were financial commitments for the patient in attending multiple appointments (55.36% of respondents reported rarely or never discussing this), indirect costs of treatment (55.36%) and self-consciousness with fixed appliance (46.42%).

#### Surgical risks

**Figure 5.**
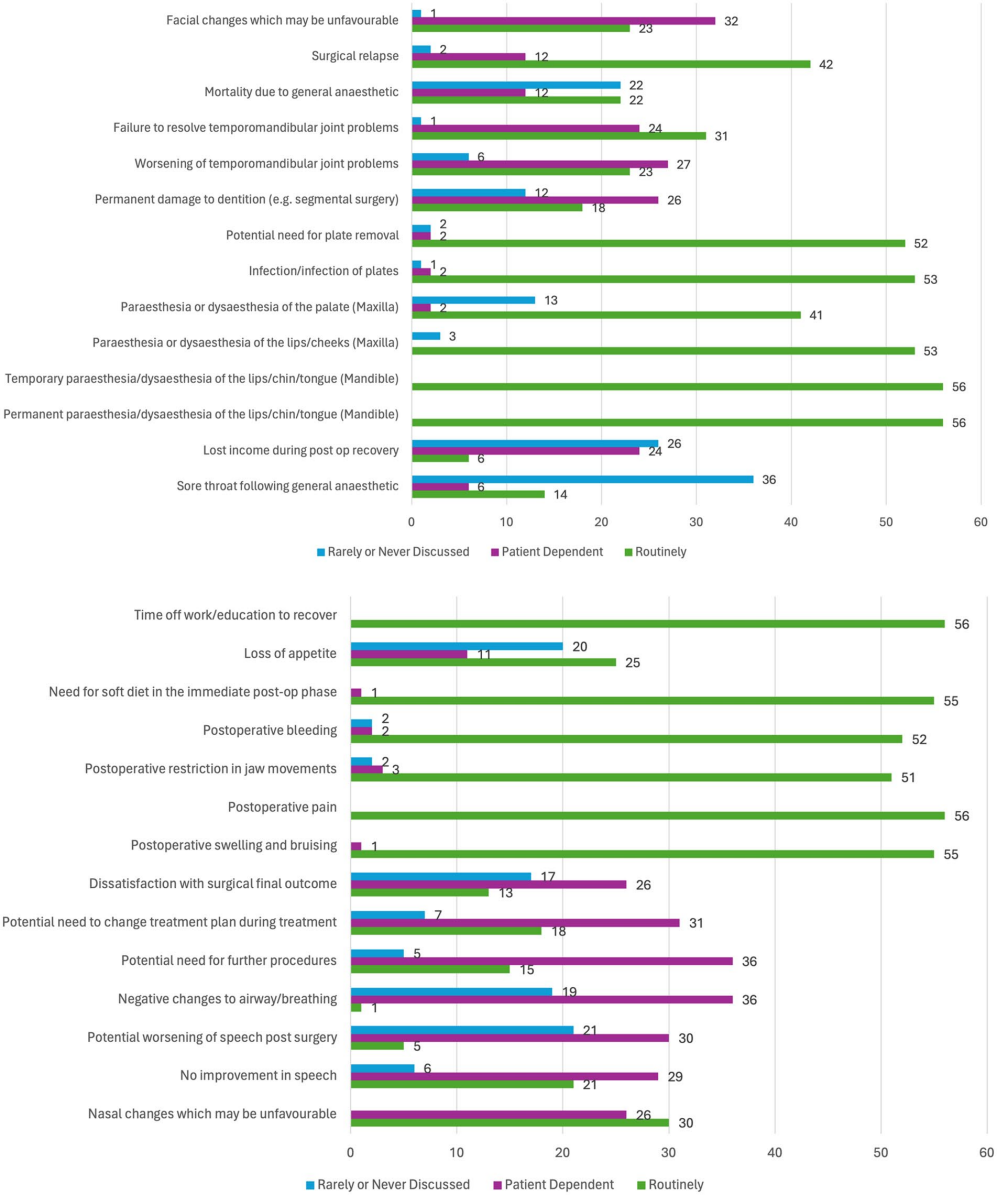
Which surgical risks and other potentially negative aspects of treatment do your team routinely discuss with patients considering/undergoing orthognathic treatment. NB: Number of responses shown.

There were four surgical risks that were ‘routinely’ discussed by all 56 respondents (100%) (Figure 5). These were permanent paraesthesia or dysaesthesia of the lips/chin/tongue (for mandibular surgery), temporary paraesthesia or dysaesthesia of the lips/chin/tongue (for mandibular surgery), postoperative pain and time off work or education to recover. A further seven responses were ‘routinely’ discussed by more than 90% of respondents. These were postoperative swelling and bruising (98.21%), postoperative bleeding (98.21%), need for soft diet in the immediate postoperative phase (98.21%), paraesthesia or dysaesthesia of the lips/cheeks (for maxillary surgery) (94.64%), infection/infection of plates (94.64%), potential need for plate removal (92.86%) and postoperative restriction in jaw movement (91.07%).

The options that were ‘rarely or never discussed’ were potential need for further procedures (64.23% of respondents reported rarely or never discussing this), negative changes to airway/breathing (64.23%) and facial changes that may be unfavourable (57.14%).

## Discussion

This study highlights the comprehensive approach taken by consultant orthodontists in preparing patients for orthognathic treatment. Patients are well-informed through nationally available resources and detailed discussions with consultant orthodontists and a MDT who discuss the potential benefits and risks of the treatment. The primary benefits discussed by respondents include improvements in occlusion, facial aesthetics and dental aesthetics. In addition, the study revealed that a significant majority of respondents report routinely discussing a wide range of orthodontic and surgical risks, with the aim of ensuring patients are thoroughly informed before proceeding with treatment.

### Clinic attendance and information format

All of the respondents reported that their patients routinely attended an orthognathic MDT clinic before commencing any active treatment, similar to the results of a UK national clinical audit where 96.93% attended an orthognathic MDT clinic before commencing treatment (Ireland et al., 2019). The majority of respondents (71.42%) attended one or two orthognathic MDTs a month. This is similar to the results of a cross-sectional questionnaire-based study carried out in England with consultant orthodontists, where 63.9% of respondents attended one or two orthognathic MDTs a month (Brannen et al., 2023).

Regarding the format of information provided, the most frequently selected responses were verbal information (96.43%), the BOS ‘*Your Jaw Surgery*’ online resource (92.86%) and BOS information leaflets in hard copy or digital format (85.71%). With an increasing focus on sustainability, the proportion of clinicians using digital information leaflets via a QR code is also likely to increase over the next few years.

These findings align with how patients seek information. In a qualitative study with prospective orthognathic patients by Wade et al. (2025), the majority of participants reported that they sought traditional information resources such as formal consultation with a professional and verbal sharing of information, before exploring further information online.

It appears to be common practice to provide patients with information in a variety of formats. A literature review by Ikeda (2011) regarding orthodontic and orthognathic patients identified several studies that investigated the effectiveness of different modalities in presenting treatment information. The results were inconsistent but showed that there may be some benefit to presenting information in different formats (Kang et al., 2009; Thickett and Newton, 2006; Thomson et al., 2001; Wright et al., 2010). Information retention has been shown to be improved with the provision of an audiovisual presentation supplemented with written information methods or through the use of mind maps (Ahn et al., 2019). The importance of this is that a multi-sensory approach helps reinforce the information being presented, simplifies complex information and enhances memory retention (Ahn et al., 2019).

Nearly all respondents (98.21%) said they used nationally available resources produced by the BOS, with the most commonly used resource being the BOS ‘*Your Jaw Surgery*’ online resource. Interestingly, similar responses were received for paper information leaflets and digital information leaflets. The use of a QR code may be lower due to familiarity of using paper leaflets or due to concerns that patients may not access the leaflets if an additional step is required. By using nationally available resources, there is a consistent baseline level of information being delivered to patients that can then be personalised to individual patients. In the context of Montgomery *vs* Lanarkshire, valid consent requires both voluntariness and competence. Simply providing information is not enough; the patient must fully understand the information in whatever format it is given and it should be tailored to the individual.

### Useful future resources

Regarding resources and forms of support that might be considered useful for the future, the most commonly selected response was discussion with a past patient, with 73.21% of respondents saying they thought this would be useful. Some units provide this through a patient information clinic; however, there are only a very small number of these clinics currently running in the UK. The opportunity for potential patients to speak with someone who has been through treatment and can articulate the benefits and downsides from a patient perspective may be very beneficial.

Support from a mental health professional as part of the multidisciplinary team on an orthognathic clinic (71.42%) or within the hospital by referral (58.92%) were both felt to be useful. However, this can be difficult, as it requires funding to be available to set up such a service. Indeed, one unit in northeast London took several years to successfully establish their service (Casey et al., 2021). In a UK-based questionnaire study of orthodontic consultants, a low number of respondents had psychological support available during their orthognathic clinic or a designated referral pathway in place to access support (Brannen et al., 2023). Alongside this, not every hospital may have ready access to internal psychiatric services; however, there are local services available via self-referral, such as NHS Talking Therapies (formally known as Improving Access to Psychological Therapies [IAPT]), or through general medical practitioner referral. Difficulties can arise when support is not tailored specifically to the complexities of orthognathic treatment, which is why it is preferable to have dedicated mental health support.

A patient decision aid was selected as being potentially useful by 35 (62.5%) respondents. The authors are not currently aware of any readily available decision aids for orthognathic treatment.

### Benefits

If patients wish to undertake elective treatment, there must be benefits to undergoing that procedure for outcomes to be successful. In the present study, the benefits of orthognathic treatment that were ‘routinely discussed’ with patients were improvement in occlusion (92.86%), improvement in facial aesthetics (91.07%) and improvement in dental aesthetics (83.93%). Previous studies have shown that a common motivator for undertaking orthognathic treatment is to improve facial aesthetics (Murphy et al., 2011; Rustemeyer and Gregersen, 2012), and patients often wish to improve function (Baherimoghaddam et al., 2016). A recent multicentre longitudinal study carried out in Chile reported that 60% of patients reported both function and aesthetics as being important motivators for orthognathic surgery (Duarte et al., 2022). Interestingly, in their study, only 2% of patients reported aesthetics alone as their motivation for treatment.

In the present study, benefits that were ‘rarely or never discussed’ included improvement in swallowing, improvement in TMJ pain and improvement in airway/breathing issues. These are areas that have proven to be controversial for orthodontists and oral and maxillofacial surgeons. Al-Moraissi et al. (2017) conducted a systematic review of TMJ dysfunction and orthognathic surgery and concluded that orthognathic surgery was associated with a decrease in TMJ dysfunction symptoms for many patients who had symptoms before surgery; however, it created symptoms in a smaller group of patients who were asymptomatic before surgery. The difficulty for clinicians when discussing TMJ problems with patients is that this systematic review concluded that neither the presence of preoperative TMJ dysfunction symptoms or the type of jaw deformity identified which patients’ symptoms would improve, remain the same or worsen after surgery. Therefore, it is unpredictable how an individual patient may respond to treatment, and patients should be counselled appropriately. Certain patients may have improvements in breathing and a reduction in symptoms if they experience obstructive sleep apnoea (OSA) (Giralt-Hernando et al., 2019); however, in the present study, respondents were asked to exclude patients with OSA and only consider benefits they discuss when undertaking routine orthognathic surgery. As a result, it was expected that fewer respondents would discuss improvements in breathing or airway issues. A recent study in Sweden found that, for orthognathic patients who did not have preoperative airway or breathing concerns, any changes in upper airway volume did not result in improvements in oral health-related quality of life, as measured using the Oral Health Impact Profile-49 (Pellby and Bengtsson, 2024).

### Orthodontic risks

A team in Cardiff conducted a Delphi study to gain a professional consensus on what orthodontic risks should be discussed with patients (Perry et al., 2021). This study was for conventional orthodontic treatment, but it is anticipated that many of the risks discussed would be the same for orthognathic treatment. There were 10 risks on which they reached consensus that should be discussed as part of consent for orthodontic treatment: demineralisation, relapse, root resorption, pain, gingivitis, ulceration, appliances breaking, failed tooth movements, treatment duration and consequences of no treatment. The present study did not include options for failed tooth movements and the consequences of no treatment, which Perry et al. (2021) included. However, it is reassuring that the other eight risks are discussed routinely by the majority of respondents. These are risks that can occur relatively commonly for orthodontic patients, such as pain, gingivitis and demineralisation, and it is important that orthodontists discuss these risks in detail with their patients during the consent process and throughout treatment. The options which respondents said they ‘rarely or never discussed’ were financial commitment (for the patient) in attending multiple appointments, indirect costs of treatment and self-consciousness with fixed appliances. Although these issues are commonly encountered by patients, fewer respondents discussed them routinely. It is interesting that these downsides of treatment are all about impact on patients outside of actual clinical appointments. Consideration could be given to including these in future nationally developed resources.

### Surgical risks

Orthognathic surgery involves significant risks that must be communicated to patients during the informed consent process. Informed consent is crucial, especially for elective treatments. However, there does not appear to be a professional consensus study that has explored these risks comprehensively. In the present study, consultant orthodontists selected four surgical risks that are routinely discussed in their hospitals: permanent and temporary paraesthesia or dysaesthesia of the lips, chin or tongue (for mandibular surgery); postoperative pain; and time off work or education for recovery. In addition, seven risks were discussed by over 90% of respondents, including postoperative swelling and bruising, bleeding, the need for a soft diet postoperatively, paraesthesia or dysaesthesia of the lips or cheeks (for maxillary surgery), infection, potential need for plate removal and postoperative restriction in jaw movement.

There appears to be good consensus on the common risks and complications that may be experienced by patients. However, this study did not determine the timing of discussions and if the discussions were with other members of the team, such as oral and maxillofacial surgeons. The orthodontic risks may be repeated during the treatment journey; however, surgical risks may be discussed less frequently and may not be as readily recalled by patients. A study by Brons et al. (2009) found that patients recalled only 40% of the risks and complications discussed before surgery. Several reviews have examined orthognathic surgery complications. Jędrzejewski et al. (2015) identified that the most common surgical complications were nerve injury/sensitivity alteration (50%), TMJ disorders (13.64%), haemorrhage (9.09%), hearing problems (6.82%) and infection (6.82%). Lower incidences were found for nerve damage (12.1%), infection (3.4%), TMJ disorders (2.1%) and haemorrhage (1.4%) by Sousa and Turrini (2012). Nerve paraesthesia, particularly in the mandible, is a significant risk, with most cases resolving within 1 year, although a small proportion of patients experience problems beyond this period. Hanzelka et al. (2011) found that approximately 3% of patients experience paraesthesia 1 year postoperatively, with older patients at higher risk (Verweij et al., 2016).

Risks that were identified as being rarely discussed by the respondents include the potential need for further procedures, negative changes to airway/breathing and unfavourable facial changes. These outcomes could lead to patient dissatisfaction if not adequately communicated.

### Limitations

The present study does have limitations. The response rate of 28% is low; a recent meta-analysis found that the average online survey response rate is now 44.1%, but the use of incentives did not increase the response rate (Wu et al., 2022). The responses were self-reported, which is a potential limitation in any study of this type. Recall bias is also a possible limitation in any questionnaire-based study of this type, but respondents will be providing this information on a routine basis so are likely to accurately remember information that is given. It should also be noted that the results are limited to consultant orthodontists working in the UK and RoI, with the majority of responses from those working in England, which limits the generalisability to other countries worldwide, as working practices and dissemination of information with patients may differ.

### Future research

The views of oral and maxillofacial surgeons were not included in this study, and this is important to explore in future research. There is a need to support the findings of this study with the perspectives of oral and maxillofacial surgeons who work on MDT clinics alongside orthodontists and also to gain patient perspectives.

## Conclusion

- Those who responded to the study reported that patients considering orthognathic treatment routinely attended an orthognathic MDT clinic before commencing active treatment.
- Information is provided with nationally available resources and consultant orthodontists routinely discuss common benefits and risks with prospective patients to help them make informed decisions regarding their care.
- The most commonly discussed benefits of orthognathic treatment were improvement in occlusion, improvement in facial aesthetics and improvement in dental aesthetics.
- There were nine orthodontic risks and 11 surgical risks that were routinely discussed by more than 90% of respondents.

## Supplemental Material

sj-docx-1-joo-10.1177_14653125251391432 – Supplemental material for Information provision for orthognathic treatment by consultant orthodontists in the United Kingdom and Republic of Ireland: A questionnaire-based study Supplemental material, sj-docx-1-joo-10.1177_14653125251391432 for Information provision for orthognathic treatment by consultant orthodontists in the United Kingdom and Republic of Ireland: A questionnaire-based study by Robert SD Smyth, Fiona S Ryan, Sophy K Barber and Susan J Cunningham in Journal of Orthodontics

sj-docx-2-joo-10.1177_14653125251391432 – Supplemental material for Information provision for orthognathic treatment by consultant orthodontists in the United Kingdom and Republic of Ireland: A questionnaire-based study Supplemental material, sj-docx-2-joo-10.1177_14653125251391432 for Information provision for orthognathic treatment by consultant orthodontists in the United Kingdom and Republic of Ireland: A questionnaire-based study by Robert SD Smyth, Fiona S Ryan, Sophy K Barber and Susan J Cunningham in Journal of Orthodontics
