# Supplementary material for: Information provision for orthognathic treatment by consultant orthodontists in the United Kingdom and Republic of Ireland: A questionnaire-based study
Source: J Orthod. 2025 Nov 26;53(1):9–19. doi: 10.1177/14653125251391432 (PMC12999983; doi:10.1177/14653125251391432)
Supplement: sj-docx-1-joo-10.1177_14653125251391432 – Supplemental material for Information provision for orthognathic treatment by consultant orthodontists in the United Kingdom and Republic of Ireland: A questionnaire-based study [file sj-docx-1-joo-10.1177_14653125251391432.docx]

**“A national questionnaire-based study assessing information provision for orthognathic treatment”**

Screening Q. *Do you personally treat orthognathic patients in your hospital? If not, we appreciate your interest but the questionnaire relates specifically to those clinicians involved in the management of orthognathic patients.*

Yes

No

If answer is no – questionnaire skips to end and exits

# Section 1. About You

1.1. *Where is your main place of work?*

England

Scotland

Wales

Northern Ireland

Republic of Ireland

1.2. *How many years have you practiced as a Consultant Orthodontist? Please enter a numerical value and round up or down to the nearest year e.g. 10*

Enter number in textbox

1.3. *Do your orthognathic patients routinely attend an orthognathic multidisciplinary clinic prior to commencing any active treatment?*

Yes

No

Comments box

|  |
| --- |

1.4. *How many orthognathic multidisciplinary clinics do you attend each month as part of your role as a Consultant Orthodontist? Enter a numerical value e.g. 4, if you do not personally attend an orthognathic multidisciplinary clinic please enter 0*

Enter in textbox

|  |
| --- |

**Section 2: When do you provide information to prospective orthognathic patients?**

2.1. *At which appointment do you* ***first*** *provide an overview of the stages and likely timescales of orthognathic treatment? Please tick one option only*

Initial new patient consultation

An orthodontic appointment where a detailed assessment and full records are taken

First multidisciplinary clinic before any treatment starts

At the time of orthodontic consent

Other – please specify below

|  |
| --- |

2.2. At which appointment(s) *do you provide* ***detailed*** *information allowing someone to make a fully informed decision about whether to proceed with treatment? Tick all that apply*

Initial new patient consultation

An orthodontic appointment where a detailed assessment and full records are taken

Patient information clinic attended by groups of patients and friends/family

First multidisciplinary clinic before any treatment starts Orthodontic consent

Other – please specify

|  |
| --- |

**Section 3: What format of information do you provide?**

3.1. *What type of information do you provide or recommend to patients considering orthognathic treatment? Please tick all that apply*

Verbal information

Local Hospital/Trust Information leaflet

BOS Information leaflet(s)

BOS ‘*Your Jaw Surgery*’ online resource

Access to a patient information clinic attended by groups of patients and friends/family

Links to recommended YouTube Videos

Alternative recommended websites/web resources

Other (please specify)

|  |
| --- |

3.2. *If you recommend specific websites, blogs or YouTube videos, please give details below including a link if possible (N.B. The link to the BOS ‘Your Jaw Surgery’ website is not required)* Enter textbox (can be left blank if N/A)

|  |
| --- |

3.3. *Does your Hospital/Trust unit run a patient information clinic where multiple patients and friends/family attend together to receive information from the orthognathic team?*

Yes (Skip to Q3.4)

No (Skip to Q4.1)

3.4. *As you responded 'Yes', we would be grateful if you could leave your hospital name below where you provide a patient information clinic. (Please note: We are asking this question purely to avoid double counting responses to some questions from individual units, all answers will be treated in the strictest confidence and hospital names will not be used in any report)*

Enter in textbox

|  |
| --- |

3.5. *At what stage do patients generally attend this patient information clinic?*

After an initial new patient consultation but before taking orthodontic records

After taking orthodontic records but before their first multidisciplinary clinic

After their first multidisciplinary clinic but before active treatment commences

After their first multidisciplinary clinic and after active treatment commences Any other time point -please specify

|  |
| --- |

# Section 4: Use of National resources

4.1. *Do you utilise nationally available resources from the British Orthodontic Society?*

Yes

No *(skip logic to 4.3)*

4.2. *Which resources do you routinely use with orthognathic patients?*

Hard copy information leaflets

Digital information leaflets via QR code

BOS ‘*Your Jaw Surgery*’ online resource

Other (please specify)

|  |
| --- |

4.3. *Which of the following do you think are/would be useful if they were available to support patients during the decision-making process? Tick all that apply*

A patient decision aid (PDA - a tool to *support shared decision making by ensuring that treatment, care and support options are explicit)*

Support from a mental health professional (psychiatrist/psychologist) as part of the multidisciplinary team on your orthognathic clinic

Support from a mental health professional (psychiatrist/psychologist) within your Trust by referral

Discussion with a past patient(s)

Access to a patient information clinic attended by multiple patients and friends/family

Decision coaching from a trained professional (A decision coach is a trained health care provider who is non-directive and provides support understanding the options and healthcare information)

I do not think any of the above are necessary

Other (please specify)

|  |
| --- |

4.4. *At what stage in the patient’s orthognathic journey do you think decision support tools, for example such as those listed in question 4.3, would be most helpful? Tick all that apply*

At the new patient assessment

Before their first multidisciplinary clinic (During the records phase)

During the first multidisciplinary clinic

After the first multidisciplinary clinic but before active treatment commences

After the first multidisciplinary clinic and after active treatment commences

Other (please specify)

|  |
| --- |

4.5. *Do you think it would be useful to develop further methods of information provision for orthognathic patients?*

Yes

Please enter suggestions

|  |
| --- |

No

# Section 5: Risks and benefits discussed by your team for patients considering conventional orthognathic treatment (excluding Cleft/Craniofacial/OSA/TMJ surgery)

5.1. *Which* ***orthodontic*** *risks and other potential negative aspects of treatment do your team routinely discuss with patients considering/undergoing orthognathic treatment? Please give a response for each item*

| **Orthodontic risks and other potential negative aspects of treatment** | Routinely | Patient dependent | Rarely or never discussed |
| --- | --- | --- | --- |
| Duration of treatment |  |  |  |
| Time commitment for multiple appointments |  |  |  |
| Financial commitment for multiple appointments |  |  |  |
| Indirect costs of treatment |  |  |  |
| Breakages resulting in additional appointments and/or extended treatment duration |  |  |  |
| Pain and discomfort |  |  |  |
| Soft tissue trauma/ulceration |  |  |  |
| Dietary limitations |  |  |  |
| Increased need to focus on oral hygiene/ Increased time toothbrushing |  |  |  |
| Gingivitis |  |  |  |
| Gingival recession |  |  |  |
| Decalcification |  |  |  |
| Root resorption |  |  |  |
| Severe root resorption |  |  |  |
| Loss of vitality of teeth |  |  |  |
| Early termination of treatment due to poor compliance |  |  |  |
| Potential need to change treatment plan during treatment |  |  |  |
| Self consciousness with fixed appliances |  |  |  |
| Potential worsening of facial and dental aesthetics pre-surgery |  |  |  |
| Orthodontic relapse |  |  |  |
| Long-term commitment to retainer wear |  |  |  |
| Maintenance costs of long-term retainer wear |  |  |  |
| Dissatisfaction with orthodontic final outcome |  |  |  |
| Other (please specify) |  |  |  |
|  |  |  |  |
|  |  |  |  |
|  |  |  |  |
|  |  |  |  |

If you discuss any other orthodontic risks or potential negative aspects of treatment could you enter them below

5.2. If you have any further comments please include them belo

***5.3.*** *Which* ***surgical*** *risks and other potential negative aspects of treatment do your team routinely discuss with patients considering/undergoing orthognathic treatment? Please give a response for each item*

| **Surgical risks and other potential negative aspects of treatment** | Routinely | Patient dependent | Rarely or never discussed |
| --- | --- | --- | --- |
| Sore throat following general anaesthetic |  |  |  |
| Mortality due to general anaesthetic |  |  |  |
| Postoperative swelling and bruising |  |  |  |
| Postoperative pain |  |  |  |
| Postoperative restriction in jaw movements |  |  |  |
| Postoperative bleeding |  |  |  |
| Need for soft diet in the immediate post-op phase |  |  |  |
| Loss of appetite |  |  |  |
| Time off work/education to recover |  |  |  |
| Lost income during post op recovery |  |  |  |
| Permanent paraesthesia or dysaesthesia of the lips/chin/tongue (for mandibular surgery) |  |  |  |
| Temporary paraesthesia or dysaesthesia of the lips/chin/tongue (for mandibular surgery) |  |  |  |
| Paraesthesia or dysaesthesia of the lips/cheeks (for maxillary surgery) |  |  |  |
| Paraesthesia or dysaesthesia of the palate (for maxillary surgery) |  |  |  |
| Infection/infection of plates |  |  |  |
| Potential need for plate removal |  |  |  |
| Permanent damage to dentition (e.g. segmental surgery) |  |  |  |
| Worsening of temporomandibular joint problems |  |  |  |
| Failure to resolve temporomandibular joint problems |  |  |  |
| Surgical relapse |  |  |  |
| Facial changes which may be unfavourable |  |  |  |
| Nasal changes which may be unfavourable |  |  |  |
| No improvement in speech |  |  |  |
| Potential worsening of speech post surgery |  |  |  |
| Negative changes to airway/breathing |  |  |  |
| Potential need for further procedures |  |  |  |
| Potential need to change treatment plan during treatment |  |  |  |
| Dissatisfaction with surgical final outcome |  |  |  |
| Other (please specify) |  |  |  |
|  |  |  |  |
|  |  |  |  |
|  |  |  |  |
|  |  |  |  |
|  |  |  |  |

If you discuss any other **surgical** risks or potential negative aspects of treatment could you enter them below

|  |
| --- |

5.4. If you have any further comments please include them below

|  |
| --- |

5.5. *Which of the following* ***benefits*** *do your team discuss with patients considering/undergoing orthognathic treatment? Please give a response for each item*

| **Benefits of treatment** | Routinely | Patient  Dependent | Rarely or never discussed |
| --- | --- | --- | --- |
| Improvement in occlusion |  |  |  |
| Improvement in chewing and masticatory function |  |  |  |
| Improvement in biting |  |  |  |
| Improvement in smile |  |  |  |
| Improvement in facial aesthetics |  |  |  |
| Improvement in dental aesthetics |  |  |  |
| Improvement in self esteem |  |  |  |
| Increased self confidence |  |  |  |
| Reduced risk of dental trauma |  |  |  |
| Improvement in speech |  |  |  |
| Improvement in swallowing |  |  |  |
| Improvement in temporomandibular joint pain |  |  |  |
| Improvement in airway/breathing issues |  |  |  |
| Improvement in quality of life |  |  |  |
| Other (please specify) |  |  |  |
|  |  |  |  |
|  |  |  |  |
|  |  |  |  |
|  |  |  |  |
|  |  |  |  |

If you discuss any other **benefits** of treatment could you enter them below

|  |
| --- |

5.6. If you have any further comments please include them below

|  |
| --- |

# Section 6: Contact regarding leaflets provided

A secondary objective of this study is to collect copies of information leaflets provided, particularly those that are developed locally rather than national resources, and to explore the content of these leaflets. Please be aware that individual leaflets will not be replicated. This information will be used to help inform the development and testing of decision support tools.

**If you are happy to share your local information leaflets with the team undertaking this project please can you forward them to either of the following;**

Email:

Postal Address:
