# Supplementary material for: Information provision for orthognathic treatment by consultant orthodontists in the United Kingdom and Republic of Ireland: A questionnaire-based study
Source: J Orthod. 2025 Nov 26;53(1):9–19. doi: 10.1177/14653125251391432 (PMC12999983; doi:10.1177/14653125251391432)
Supplement: sj-docx-2-joo-10.1177_14653125251391432 – Supplemental material for Information provision for orthognathic treatment by consultant orthodontists in the United Kingdom and Republic of Ireland: A questionnaire-based study [file sj-docx-2-joo-10.1177_14653125251391432.docx]

**Supplementary Figure 2:** Strobe checklist

| **Recommendation** | **Item No** | **Page Number** |
| --- | --- | --- |
| Title and abstract | 1 | 1 |
| **Introduction** |  |  |
| Background/rationale | 2 | 2 |
| Objectives | 3 | 3 |
| **Methods** |  |  |
| Study design | 4 | 3 |
| Setting | 5 | 3 |
| Participants | 6 | 4 |
| Variables | 7 | 3 |
| Data sources/measurement | 8 | 3-4 |
| Bias | 9 | N/A |
| Study size | 10 | 4 |
| Quantitative variables | 11 | N/A |
| Statistical methods | 12 | 4 |
| **Results** |  |  |
| Participants | 13 | 4 |
| Descriptive data | 14 | 4 |
| Outcome data | 15 | 4-7 |
| Main results | 16 | 4-7 |
| Other analyses | 17 | N/A |
| **Discussion** |  |  |
| Key results | 18 | 7 |
| Limitations | 19 | 11 |
| Interpretation | 20 | 7-11 |
| Generalisability | 21 | 11 |
| **Other information** |  |  |
| Funding | 22 | 12 |
